# Not your mean green: beyond averages in mapping socio-spatial inequities in urban greenery for smart cities

**DOI:** 10.1140/epjds/s13688-026-00627-4

**Published:** 2026-02-09

**Authors:** Jenny Martinez, Javier Argota Sánchez-Vaquerizo, Sachit Mahajan

**Affiliations:** 1 Institute of Sustainable Development (INE), School of Engineering (SoE), Zurich University of Applied Sciences (ZHAW), Technoparkstrasse 2, Winterthur, 8400 Switzerland; 2 Computational Social Science, ETH Zurich, Stampfenbachstrasse 48, Zurich, 8092 Switzerland

**Keywords:** Urban greenery, Environmental justice, Smart cities, Spatial inequality, Canopy height, Colombia

## Abstract

As data-driven “smart city” agendas expand across Latin America, most urban performance metrics remain focused on infrastructure, connectivity, and aggregate efficiency, often neglecting who truly benefits. Urban greenery, a vital determinant of health and climate resilience, is one such blind spot. While some frameworks now consider “green space,” they do so at a coarse, citywide scale, overlooking how access is distributed across neighborhoods and social groups. This obscures critical equity gaps, particularly in cities marked by deep socio-spatial segregation. In this study, we develop a fully reproducible geospatial pipeline that integrates high-resolution canopy height models, public park data, gridded population estimates, and socioeconomic strata to assess how greenery is distributed, not just how much exists. Applied to Bogotá and Medellín, the method reveals stark disparities: population-weighted canopy coverage rises significantly between the lowest and highest strata, while access to public parks also shows measurable inequality, especially in high-density, underserved neighborhoods. These inequities persist despite progressive greening policies, revealing the limits of optimization when legacy segregation is ignored. Our open-source pipeline enables finer-grained, justice-oriented audits that go beyond averages to identify where greenery and its benefits are most lacking. By enabling fine-grained equity assessments, this approach underscores the importance of greenery distribution, not just quantity, as a critical indicator for inclusive and equitable smart cities.

## Introduction

The global rise of the “smart city” paradigm has reshaped urban policy, promising enhanced quality of life through data-driven governance, optimized infrastructure, and digital connectivity [1–4]. Influential indices such as the Instituto de Estudios Superiores de la Empresa (IESE) Cities in Motion Index—which ranks cities across nine dimensions including economy, governance, and environment using publicly available indicators—and the Institute for Management Development (IMD) Smart City Index—which combines structural data with resident perception surveys to assess urban performance—translate this agenda into comparable scores across cities, emphasizing domains like technology adoption, economic performance, mobility, and service delivery [5, 6]. Yet, while such frameworks increasingly nod to sustainability, they typically prioritize aggregate efficiency and performance, with limited attention to justice, inclusion, or the distribution of benefits across different social groups [7–9]. Even environmental indicators (e.g., “green space”) are commonly operationalized as citywide aggregates that say little about who benefits or where access is scarce [10, 11].

Within this landscape, the IMD Smart City Index instrument is notable for asking two distinct perception questions about urban greenery: whether residents find green spaces *satisfactory*, and whether greenery ranks among their *top priorities*. These items capture sentiment and relative salience, respectively, but are reported as single city-level scores derived from small samples, limiting their ability to reveal within-city disparities. In other words, they help us understand *what people feel* in the aggregate, not *who has access* or *where* green infrastructure is lacking.

Urban greenery—trees, parks, and other vegetated spaces—has moved from amenity to essential infrastructure in contemporary urban resilience and environmental justice debates [12–14]. Vegetation mitigates urban heat islands, improves air quality, contributes to stormwater management, and supports mental and physical health [15–17]. However, access to these benefits is uneven: studies across the Global North and South show that low-income and marginalized communities typically have less canopy and poorer access to parks, with measurable consequences for heat exposure and health outcomes [18–21]. Global policy frameworks, such as the Sustainable Development Goal (SDG) 11, explicitly call for universal access to safe, inclusive, and accessible green spaces by 2030 [22], which in turn requires moving beyond citywide averages to diagnose intra-urban disparities that smart city indices rarely measure directly [23, 24].

Latin American cities, shaped by rapid urbanization and entrenched socio-spatial segregation, offer a critical vantage point for examining these distributional questions [25, 26]. In Colombia, the *estratificación socioeconómica* (Spanish for socioeconomic stratification) policy classifies neighborhoods into six *estratos* or strata (1 = lowest, 6 = highest) for pricing and planning [27, 28]. While designed to improve equity in service provision, the system has coincided with greener, cooler, and better-amenitized environments in higher-stratum areas, while lower-stratum neighborhoods often face sparse canopy and concrete-dominated streetscapes [29–31]. These patterns amplify vulnerabilities to heat and flooding [14, 32]. Recent initiatives like Medellín’s *Corredores Verdes* (Spanish for green corridors) (launched in 2017) and the city of Bogotá’s *Plan de Ordenamiento Territorial* (POT, Spanish for Comprehensive Land Use Plan) 2022–2035—explicitly aim to expand vegetation and advance environmental justice [33–35]. Yet concerns persist about implementation gaps and “green gentrification,” where greening raises property values and risks displacement [36, 37]. Empirical evidence on whether such policies narrow green inequities remains limited.

Against this regional context, IMD’s perception data provide a useful, if coarse, reference point. In the 2025 release, 65.9% of the 120 surveyed residents in Bogotá and 76.3% in Medellín ($n=120$) agreed or strongly agreed with the statement that their city’s green spaces are “satisfactory,” yet only 11.5% and 8.9%, respectively, list greenery among their top five urban priorities [6]. These figures are derived from small samples and reported as aggregate percentages of positive agreement, making them useful for gauging overall sentiment but silent on where greenery is abundant or absent and which populations are underserved within each city. This gap between perception-based indices and distributional realities motivates the approach we take here.

This study examines the distribution of urban greenery in Bogotá and Medellín through an environmental justice and spatial equity lens. We develop a fully reproducible geospatial pipeline that integrates high-resolution canopy height models (CHM) [38], gridded population estimates [39], official stratum boundaries, and OpenStreetMap (OSM) features for public green space. Our metrics explicitly weight by population to summarize *experienced* canopy coverage across socio-economic strata and quantify proximity to public parks, thereby shifting the question from “how much greenery does a city have?” to “who has access, and where are deficits concentrated?” Applied to two Colombian cities, the analysis reveals persistent “green gaps” structured by socio-economic segregation, even amid progressive greening strategies. By openly sharing data and code, we show how smart city tools can be redirected toward transparency, reproducibility, and equity-focused governance. We argue that urban greenery is an essential infrastructure feature and should be treated as a core metric for inclusive, resilient, and *truly* smart cities.

## Related work

### Beyond technocentric smart cities: the socio-ecological blind spot

The smart city paradigm has evolved over the past decade, shifting from a technocratic focus on information and communication technologies (ICT), infrastructure optimization, and economic metrics to broader considerations of sustainability and resilience [1, 2, 8]. However, critical urban scholars highlight a persistent “socio-ecological blind spot” in mainstream smart city frameworks, where social equity and environmental justice are often sidelined [7, 40, 41]. This contrasts with the notion of the city as a social construct daily shaped by collective rights and struggles [42, 43], and with calls for a democratic, inclusive, and justice-oriented digital urbanism, particularly in the case of smart cities [44]. Early critiques by Hollands [7] warned that corporate-driven agendas, led by technology vendors and urban managers, prioritize efficiency and control over inclusive governance. For example, smart city indices like ISO 37120 and 37122 by the International Organization for Standardization, the IESE Cities in Motion, and the IMD Smart City Index emphasize metrics such as public Wi-Fi coverage, traffic flow optimization, or energy efficiency but rarely investigate who benefits from these interventions [5, 6, 45].

Recent analyses confirm that equity and social inclusion remain marginal in most smart city projects [46, 47]. This gap is particularly pronounced in the Global South, where resource constraints and informal urban systems complicate the adoption of equity-focused smart city models [9]. The use of digital solutions in unequal urban contexts often exacerbates divides, favoring affluent, digitally literate groups while marginalizing low-income or less-connected communities [48–50]. Moreover, technology-driven interventions can introduce accessibility burdens, such as increased energy consumption or e-waste, which can disproportionately impact vulnerable populations.

In response, global policy frameworks like SDG 11 advocate for “inclusive, safe, resilient, and sustainable cities” that integrate technological innovation with justice-oriented goals [22]. Scholars call for smart city governance that embeds socio-ecological equity into design and evaluation, ensuring that benefits like green infrastructure access reach marginalized groups [11, 36, 51].

A review of some of the leading international smart city indices (Table 1) shows that, while all include at least one greenery-related indicator, these are typically aggregated at the city scale. The IMD Smart City Index relies primarily on perception surveys, asking residents whether green spaces are satisfactory. The IESE Cities in Motion Index uses quantitative measures such as green area per capita. The Arcadis Sustainable Cities Index integrates environmental, social, and economic pillars but reports greenery only as a percentage of city area. The City Biodiversity Index (CBI), developed under the Convention on Biological Diversity, includes 28 biodiversity-related indicators but remains a voluntary self-assessment tool. The ISO 37120 and 37122 standards provide standardised indicator definitions for city services and smart city performance, respectively, including green space metrics, but adoption and reporting remain uneven across cities. Accessibility, spatial equity, and canopy quality—critical for environmental justice—are absent. None of these indices explicitly measures tree canopy coverage, equitable park access, or the ecological services of greenery. Furthermore, none provides sub-city disaggregation, a limitation that is especially problematic in socio-spatially stratified cities. In such contexts, single citywide indicators can mask acute local deficits, rendering them invisible in global comparisons and rankings.

**Table 1:** Treatment of urban greenery in major international smart city indices. All indicators are reported at the city level; none are disaggregated within cities

Index	Availability	Accessibility	Disaggregation
IMD Smart City [6]	No quantitative area; survey-based only	No	Citywide only
IESE Cities in Motion [5]	Green area per capita; % area	No	Citywide only
Arcadis Sustainable Cities [52]	% of city area	No	Citywide only
City Biodiversity Index (CBI) [53]	28 biodiversity-related indicators (ecosystems, services, governance), incl. % area and connectivity	Partial (proximity-based only)	Citywide only
ISO 37120/37122 [54, 55]	Green area per 100,000 pop.; % city area; share of pop. within 0.5 km of open space; ICT-based monitoring (37122)	Partial (proximity-based only)	Citywide only

### Urban greening, environmental justice, and green inequity

Environmental justice scholarship has extensively documented the unequal distribution of urban greenery and its profound impacts on health, well-being, and climate resilience [12, 13, 56]. Urban vegetation—trees, parks, and green corridors—provides critical ecosystem services: cooling heat-stressed neighborhoods [57, 58], improving air quality [59, 60], managing stormwater [61, 62], and fostering recreation and social cohesion [24, 63]. Yet, studies worldwide reveal persistent green divides: low-income and marginalized communities consistently have less access to green spaces, street trees, and canopy cover compared to wealthier areas [19, 64]. For example, research found that in several U.S. cities, low-income neighborhoods have significantly lower tree canopy, correlating with higher summer temperatures, a pattern termed thermal inequity [65, 66]. These disparities are particularly stark in the Global South, where rapid urbanization and historical segregation exacerbate green inequity [67, 68]. In South Africa, green infrastructure remains unequally distributed among different races and income groups [69]. In Bogotá, low-stratum neighborhoods often lack street trees and public parks [20, 29], often falling below the World Health Organization’s recommended 9 m^2^ of green space per capita [70]. Similar patterns appear in São Paulo, where informal settlements have limited access to formal green spaces [71]. Across Latin America, most cities exhibit sharp intra-urban variations in green space access, with low-income areas disproportionately underserved [72, 73].

In response, the concept of “tree equity” has gained traction, advocating for targeted greening in underserved areas and linking canopy data to health and social outcomes [74, 75]. Community-led initiatives, such as participatory tree planting, further emphasize inclusive approaches [76]. However, greening interventions can trigger green gentrification, where new parks or green corridors increase property values, risking displacement of vulnerable residents [77, 78]. Scholars thus advocate for “just green enough” strategies—community-driven, equitable greening that prioritizes local needs without fueling gentrification [13, 79].

### Latin American urban inequality, policy, and participation

Some Latin American cities highlight socio-spatial segregation, shaped by historical patterns of informality, centralized planning, and entrenched class divides [14, 80]. In Colombia, the “estratificación socioeconómica” system formalizes these divides by classifying neighborhoods into six strata, guiding utility subsidies and urban planning [28, 81]. While intended to promote equitable service access, this system often perpetuates stigma, inequalities and prioritizes investment in higher-stratum areas, including parks and green infrastructure [20]. For example, in Bogotá and Medellín, wealthier neighborhoods boast lush street trees and accessible parks, while low-stratum areas face green deficits, exacerbating climate vulnerabilities [33, 82]. Recent policy innovations aim to address these disparities. Medellín’s Green Corridors, launched in 2017, have expanded urban greenery, targeting underserved Comunas to reduce heat [33, 34]. Bogotá’s POT 2022–2035 prioritizes equitable greening through community-driven ecological zones [35]. However, challenges like land tenure insecurity [83] in informal settlements complicate equitable implementation, risking exclusion of marginalized groups. Latin America has a long tradition of participatory urbanism, from Porto Alegre’s participatory budgeting [84–86], to Colombia’s Juntas de Acción Comunal that organize neighborhood improvements [87], and more recently, Medellín’s integrated urban projects that linked social urbanism with greening [88]. These experiences show how collective action offer potential for inclusive greening, yet questions persist about the reach and maintenance of these initiatives in lower-stratum or informal areas

### Critical urban data science and open methods

The convergence of critical urban studies and data science has revolutionized the study of urban inequalities, leveraging high-resolution spatial analytics to map green space, pollution, water levels and heat at fine scales [89–92]. Open-source tools, combining remote sensing, GIS, and civic tech, enable granular analyses of environmental disparities [93, 94], as seen in global canopy mapping and population datasets [39, 95]. Machine learning techniques further enhance these efforts, identifying patterns in urban greenery and health outcomes [96]. However, scholars caution that data and technology are not neutral: their design, access, and application must prioritize equity to avoid reinforcing biases [97]. In the Global South, where high-resolution data are often scarce, open and transparent methods are critical for inclusive urban governance. Open-source pipelines, like the one we present, democratize access to data and foster accountability, aligning with calls for “data for social good” in urban analytics [98]. By sharing replicable methods, our study contributes to this ethos, offering a framework to assess green equity in cities like Bogotá and Medellín and applicable to other cities, where data-driven insights can inform equitable policy

Our study builds on these literatures, integrating critical smart city theory, environmental justice, Latin American urban policy, and open geospatial data science. By applying and sharing a replicable, equity-focused canopy analysis, we offer both a substantive and methodological contribution to the question: what (and whom) do smarter cities truly optimize for?

#### Justification for study area selection

We selected Bogotá and Medellín as case studies for four reasons. First, Colombia’s *estratificación socioeconómica* system classifies neighborhoods into six socioeconomic strata, enabling direct linkage between greenery and socioeconomic status without proxy variables. Second, the two cities offer contrasting urban forms—Bogotá on a high-altitude plateau, Medellín in a narrow Andean valley—allowing examination of how topography and eco-geographic context shape green inequities. Third, both cities have implemented progressive greening policies (Medellín’s *Corredores Verdes*; Bogotá’s POT 2022–2035) and appear in international smart city indices, making them relevant cases for assessing whether such interventions reduce spatial inequities. Finally, the availability of open administrative and remote sensing data supports the reproducibility objectives of our pipeline.

## Methodology

**Figure 1:**
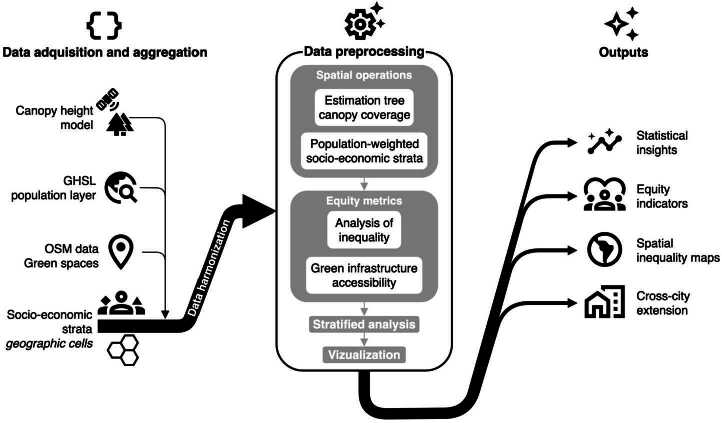
Schematic of the equity-analysis pipeline. Input layers are ingested and harmonised, to be later processed as explained in Sect. 3, producing four classes of decision-ready outputs

This study integrates high-resolution canopy height mapping, socio-economic stratification data, gridded population estimates, and public green space locations to examine the spatial distribution of urban greenery in Bogotá and Medellín. Our methodology consists of four key phases: (i) data acquisition and integration, (ii) canopy and park access estimation, (iii) population-weighted inequality analysis, and (iv) spatial and statistical testing. Figure 1 summarises the overall analytical workflow, from initial data layers to actionable outputs.

Access to green space is a multidimensional concept. Established frameworks, such as Penchansky and Thomas’s five dimensions of access and Saurman’s subsequent extension to six dimensions [99, 100], distinguish between availability (presence and supply), accessibility (geographic proximity), affordability (financial barriers), acceptability (social and cultural appropriateness), awareness (knowledge of services), and adequacy (quality and suitability). In this study, we operationalise access along two of these dimensions: (1) *availability*, measured as the proportion of tree canopy coverage within residential blocks, capturing the presence of vegetative infrastructure where people live; and (2) *accessibility*, measured as Euclidean distance from block centroids to the nearest public park, reflecting geographic proximity to formal recreational green space. We do not assess affordability, acceptability, awareness, or adequacy, as these dimensions require survey data, qualitative assessment, or fine-grained quality indicators beyond the scope of this analysis. Readers should therefore interpret our findings as characterising the spatial distribution and proximity of green infrastructure, rather than the full spectrum of barriers and enablers that shape residents’ lived experiences of urban greenery.

### Data acquisition and integration

As depicted in Fig. 1, our pipeline begins by assembling four open-access layers for Bogotá and Medellín and harmonising them at the level of official strata block polygons. These polygons are the spatial units defined by Colombian municipalities to assign each residential block to one of six socioeconomic strata (*estratos*) for utility pricing and urban planning purposes. A global canopy height model produced by Meta and the World Resources Institute provides 0.5–1 m resolution estimates of vegetation height [38]; these rasters are clipped to the built-up footprint of each city. Socio-economic context comes from municipal GeoJSON files that classify every block into one of six strata categories used for public-service pricing and planning: for Bogotá, we obtained stratification polygons from Datos Abiertos Bogotá [101], and for Medellín, from the GeoMedellín open data portal [102]. We overlay a 100 m Global Human Settlement Layer (GHSL) population grid and aggregate its counts to each polygon, thus weighting subsequent statistics by the number of residents affected.

We opted to use the GHSL gridded population estimates rather than local census microdata (e.g., Departamento Administrativo Nacional de Estadística 2018) to prioritize the transferability of this workflow. While local administrative records offer higher precision and avoid the need for dasymetric downscaling, they are frequently unavailable, outdated, or restricted in many Global South contexts. By validating our pipeline using globally standardized open data, we ensure that this equity analysis remains reproducible in cities where high-resolution local census data may not be accessible.

The spatial support for this integration is the *manzana* (Spanish for urban block), which constitutes the smallest administrative unit for census and stratification purposes. The analysis covers the entire continuous urban footprint, comprising approximately 37,400 residential blocks in Bogotá and 31,500 in Medellín. While the high resolution of the CHM (1 m) ensures that even small blocks contain sufficient pixel data for robust canopy estimation, we acknowledge that the GHSL population grid (100 m) has a coarser resolution than the smallest urban blocks. Consequently, while population assignments to individual blocks may contain interpolation noise (MAUP), our primary analysis relies on aggregating these units into broad socioeconomic strata, thereby mitigating local estimation errors through the law of large numbers.

Public green-space features—parks, gardens, playgrounds, and recreation grounds—are extracted from OSM [103], with private and natural-area tags discarded so that our distance metric reflects everyday recreational access. After checking geometry validity, all layers are transformed to a common equal-area coordinate reference system (CRS) for canopy and area calculations, and duplicated in a city-specific conformal projected CRS for distance measurements. Non-residential polygons and small artefacts are removed to avoid bias. The resulting table—linking canopy cover, population, nearest-park distance, and strata for every block—serves as the foundation for the canopy, park-access, and inequality analyses that follow.

The inclusion of two complementary data sources for green infrastructure, i.e. tree canopy and OSM public green features, aims to consider greenery not formally recorded in official inventories, which is particularly relevant in informal settlements and other areas potentially under-represented in official datasets. The integration of both green-related information provides a more comprehensive representation of urban greenery, taking into account that each component provide partially overlapping yet distinct social and ecosystem services and benefits.

### Estimation of tree canopy coverage

To quantify vegetation coverage across urban blocks, we computed the proportion of surface area in each polygon with canopy heights above a minimum threshold $h_{min}$ meters. This is based on the CHM raster clipped to polygonal boundaries. The canopy coverage $C_{p}$ for a given polygon *p* is calculated as:

1 $$ C_{p}(h_{min}) = \frac{N_{p}^{h > h_{min}}}{N_{p}^{\mathrm{valid}}} \times 100 $$

where $N_{p}^{h > h_{min}}$ is the number of pixels within polygon *p* with height values exceeding $h_{min}$, and $N_{p}^{\mathrm{valid}}$ is the total number of non-null pixels in that polygon. We performed the analysis at three different thresholds: $h_{min} = 1.5$ m, 2.0 m, and 3.0 m. The results presented in this work use $h_{min} = 2.0$ m as the primary benchmark (based on literature and sensitivity analysis results discussed later).

In cases where no valid CHM data were available for a polygon (e.g., due to shadow or cloud cover in original imagery), the canopy coverage was marked as missing.

### Population-weighted aggregation by socio-economic strata

To meaningfully compare greenery access across socio-economic groups, we aggregated canopy coverage values using population-weighted means. For each stratum *s*, the weighted average canopy coverage $\overline{C}_{s}$ is given by:

2 $$ \overline{C}_{s} = \frac{\sum \limits _{p \in S_{s}} C_{p} \cdot P_{p}}{\sum \limits _{p \in S_{s}} P_{p}} $$

where $S_{s}$ is the set of polygons in stratum *s*, $C_{p}$ is the canopy coverage of polygon *p*, and $P_{p}$ is the estimated population of *p*. This accounts for the fact that larger or denser polygons contribute more to the average experience of residents. We report $\overline{C}_{s}$ across multiple height thresholds and both cities.

We also computed the per capita canopy coverage per polygon:

3 $$ c_{p} = \frac{C_{p}}{P_{p} + \varepsilon} $$

where *ε* is a small constant to prevent division by zero. While this metric offers insight into individual-level access, we note its limitations when population data are sparse or uncertain.

### Statistical and distributional analysis of inequality

We applied a comprehensive suite of statistical, distributional, and spatial metrics to evaluate how tree canopy coverage varies across socio-economic strata in both cities. This multi-pronged approach provides insight not only into mean group differences, but also the broader patterns of inequality and spatial clustering that characterize green infrastructure access.

#### Association between socioeconomic status and canopy coverage

We quantified the monotonic association between socio-economic status and canopy coverage using Spearman’s rank correlation coefficient (*ρ*), which is robust to outliers and non-linear relationships. Here, polygons are ranked by stratum, and the correlation is computed with respect to their canopy coverage:

4 $$ \rho = 1 - \frac{6 \sum _{p=1}^{n} d_{p}^{\,2}}{n(n^{2} - 1)}, $$

where $d_{p}$ is the rank difference for polygon *p*, and *n* is the total number of polygons. A positive *ρ* indicates increasing canopy with higher socio-economic status.

#### Groupwise differences in mean canopy coverage

We tested for statistically significant differences in mean canopy coverage across the six strata using one-way ANOVA (for approximate normality and homoscedasticity) and the Kruskal–Wallis *H* test when these assumptions were not met. Levene’s test was used to assess variance homogeneity. Tukey’s HSD test was used for post-hoc pairwise comparisons, with differences visualized as heatmaps in the Results section.

#### Categorical inequality across canopy classes

Canopy coverage was discretized into Low ($<10\%$), Medium (10–30%), and High ($\geq 30\%$) classes, reflecting established ecological thresholds for urban greening [104]. We applied a Pearson $\chi ^{2}$ test to assess independence between canopy class and stratum, using the standard statistic:

5 $$ \chi ^{2} = \sum _{i=1}^{k} \sum _{j=1}^{m} \frac{(O_{ij} - E_{ij})^{2}}{E_{ij}}, $$

where $O_{ij}$ is the observed frequency of polygons in stratum *i* and canopy class *j*, $E_{ij} = (\sum _{j} O_{ij} \cdot \sum _{i} O_{ij}) / N$ is the expected frequency under the null hypothesis of independence, *N* is the total number of polygons, $k = 6$ strata, and $m = 3$ canopy classes.

#### Distributional inequality metrics

To summarise and decompose the broader distributional pattern of greenery, we computed three complementary indices, all weighted by polygon population.

*Canopy Concentration Index (CCI).* The CCI ranks polygons by stratum and measures the area between the concentration curve and the equity line:

6 $$ \mathrm{CCI}=2\!\left (\tfrac{1}{2}-\!\sum _{i=1}^{n-1}\tfrac{1}{2} \left (L_{i}+L_{i+1}\right )\!\left (u_{i+1}-u_{i}\right )\right ), $$

where $u_{i}$ and $L_{i}$ are, respectively, the cumulative population and cumulative canopy shares at rank *i*. $\mathrm{CCI}=0$ indicates perfect equity; positive values indicate a pro-rich neighborhoods skew [105, 106].

*Population-weighted Gini coefficient.* We use the covariance form of the weighted Gini, which is scale-invariant and population-consistent:

7 $$ G= \frac{\sum _{i=1}^{n}\sum _{j=1}^{n}w_{i}\,w_{j}\,|C_{i}-C_{j}|}{2\,\mu \,\sum _{i=1}^{n}w_{i}}, $$

where $C_{i}$ and $C_{j}$ denote canopy coverage for polygons *i* and *j*, $w_{i}$ and $w_{j}$ are their respective population weights ($P / \sum P$), and $\mu =\sum w_{i} C_{i}$ is the population-weighted mean [107]. To avoid undefined logs in subsequent indices, zero-canopy polygons are perturbed by an infinitesimal $\varepsilon =10^{-6}$.

*Theil T Index and decomposition.* We use the Theil T Index because, unlike the Gini coefficient, it is additively decomposable: total inequality can be partitioned into a between-group component (attributable to differences across strata) and a within-group component (residual variation within each stratum). This property allows us to quantify the share of canopy inequality explained by socioeconomic stratification. The decomposable Theil T is

8 $$ T_{T}= \frac{\sum _{i=1}^{n}w_{i}\left (\tfrac{C_{i}}{\mu}\right )\ln \!\left (\tfrac{C_{i}}{\mu}\right )}{\sum _{i=1}^{n}w_{i}}, $$

with the between-group component

9 $$ T_{\mathrm{between}}= \frac{\sum _{g=1}^{G}w_{g}\left (\tfrac{\mu _{g}}{\mu}\right )\ln \!\left (\tfrac{\mu _{g}}{\mu}\right )}{\sum _{g=1}^{G}w_{g}}, $$

where $w_{g}$ and $\mu _{g}$ are the population share and mean canopy of group *g*. The within-group share is $T_{\mathrm{within}} = T_{T} - T_{\mathrm{between}}$. All log terms use $C_{i}+\varepsilon $ with the same *ε* as above.

#### Spatial clustering of canopy coverage

We assessed spatial clustering with Moran’s *I* on polygonal units using a row–standardized Queen contiguity weights matrix. Moran’s *I* is

10 $$ I \;=\; \frac{n}{W}\; \frac{\sum _{i}\sum _{j} w_{ij}\,(x_{i}-\bar{x})(x_{j}-\bar{x})}{\sum _{i}(x_{i}-\bar{x})^{2}}, $$

where $x_{i}$ is canopy coverage for polygon *i*, $w_{ij}$ are spatial weights, and $W=\sum _{i}\sum _{j} w_{ij}$. In some cases, the Queen contiguity graph yielded more than one connected component, which can prevent a valid global autocorrelation calculation. When this occurred, we adopted a *k*–nearest neighbors (KNN) fallback with $k=8$, row–standardized, to ensure a fully connected weights graph while maintaining a local neighborhood scale (i.e., capturing the immediate surroundings in a gridded street network). Statistical significance was assessed by 999 Monte Carlo permutations.

#### High-risk polygon identification

Drawing on urban environmental health literature [65], polygons were flagged as high-risk if they simultaneously met the criteria of $C_{p} < 10\%$ canopy and $P_{p} > 500$ population. The 10% canopy threshold corresponds to the lower boundary of our categorical analysis and aligns with urban forestry research identifying minimal tree cover thresholds [104]. The population threshold of 500 residents was selected to focus on areas where green space scarcity affects substantial numbers of people. This value is high enough to target meaningful concentrations of residents, yet low enough to identify a sufficient number of polygons for analysis. The robustness of this choice was evaluated through a sensitivity analysis with alternative thresholds (400 and 600 residents), presented in the Results. This dual criterion identifies zones where large numbers of residents experience acute green space scarcity, representing priority areas for environmental equity interventions.

#### Robustness to canopy height threshold

To confirm the robustness of all findings, we repeated the entire analysis at alternative canopy thresholds ($h_{\min}=1.5$ m and 3.0 m, in addition to 2.0 m). The observed inequality patterns and stratified group ordering remained consistent, supporting the validity of our conclusions.

### Distributional analysis of public park access using OSM data

To complement our analysis of tree canopy, we also examined how access to public green spaces is distributed across socio-economic groups using data from OSM. We focused on areas specifically tagged as parks, gardens, playgrounds, and recreation grounds, and we excluded features such as forests, private gardens, and other natural or private areas. This selection was made to concentrate on public urban parks and recreational spaces that are most relevant for daily community use and urban policy.

For each residential block centroid, we measured the straight-line (Euclidean) distance to the nearest public park. This gives a simple, intuitive indicator of how close each neighborhood is to formal green space.

To assess inequality in park access, we calculated the Gini coefficient and the Theil index, using the same population-weighted methods described earlier for canopy coverage. Here, these metrics are applied to the distribution of nearest-park distances, providing a summary of how evenly access to parks is shared within and between socio-economic strata. All results are reported separately by stratum, allowing for direct comparison with the canopy-based findings.

This approach complements the ecological greenness measures by highlighting spatial disparities in proximity to public green spaces.

## Results

### Sensitivity analysis

To ensure our findings were not driven by the selection of the primary canopy-height threshold ($h_{\min}=2.0\,\mathrm{m}$), we compared population-weighted mean canopy coverage at $1.5\,\mathrm{m}$, $2.0\,\mathrm{m}$, and $3.0\,\mathrm{m}$.

**Figure 2:**
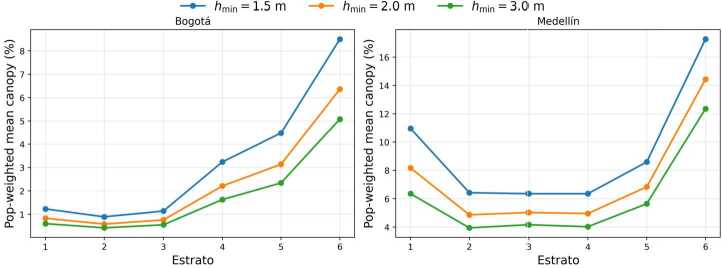
Population-weighted mean canopy coverage by *estrato* at three canopy-height thresholds ($h_{\min}=1.5,\,2.0,\,3.0$ m) for Bogotá (left) and Medellín (right). Lower thresholds yield higher absolute canopy, but the *relative* gradients across strata are stable; in both cities, stratum 6 remains the highest across thresholds

As shown in Fig. 2, changing $h_{\min}$ shifts absolute canopy values—lower thresholds yield higher coverage—but the *distributional pattern* remains stable. In Bogotá, stratum 6 consistently exhibits the highest canopy coverage across all thresholds, while strata 1–3 remain at the bottom. In Medellín, the relative gradients are similarly preserved, with stratum 6 highest and stratum 2 consistently lowest. These consistencies confirm that the observed inequality gradients are robust to parameter selection. Consequently, we present all subsequent results using the standard $h_{\min}=2.0\,\mathrm{m}$ benchmark.

### Green space inequality: canopy cover and park access

This section examines the distribution of green infrastructure across socioeconomic strata in Bogotá and Medellín, using two complementary perspectives: (1) high-resolution estimates of tree canopy coverage, and (2) spatial analysis of proximity to open green spaces derived from OSM data.

Canopy coverage serves as a proxy for broader vegetative presence and ecosystem services (e.g., shade, cooling), while green space proximity captures physical access to formal recreational green space. These two forms of greenery are shaped by distinct planning regimes and socio-spatial logics, and thus may exhibit different inequality patterns.

We analyze both cities through this dual lens, applying descriptive statistics, inferential tests, and spatial inequality metrics to reveal the extent, structure, and drivers of urban green space inequality.

#### Bogotá: extreme environmental stratification

**Figure 3:**
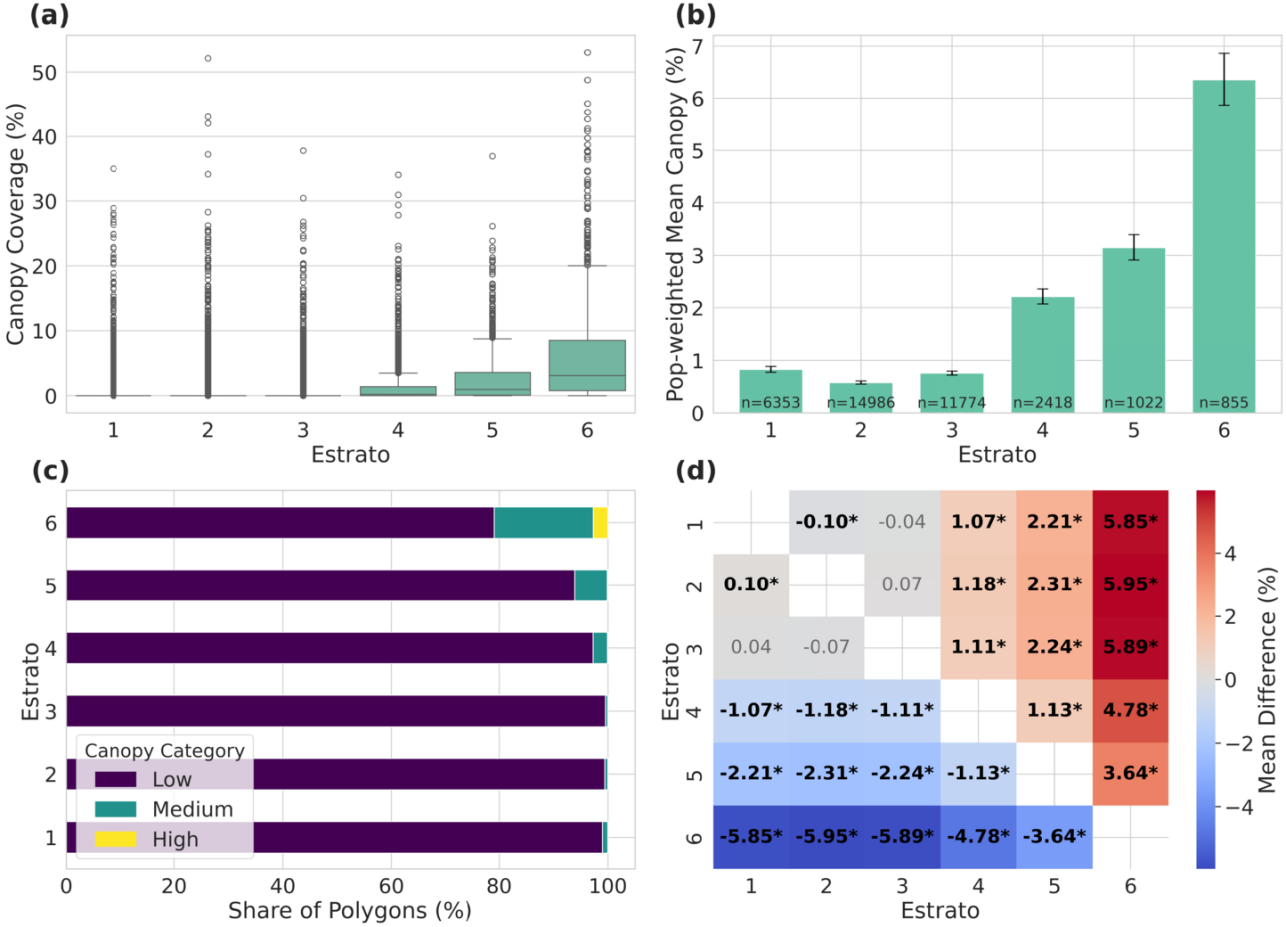
Distribution of tree canopy cover by socioeconomic stratum in Bogotá. (a) Boxplots of canopy coverage (%) by stratum, showing medians, interquartile ranges, and outliers; (b) Mean canopy coverage with 95% confidence intervals by stratum, with *n* indicating the number of spatial units in each group; (c) Stacked bars showing the percentage of area in each canopy class (Low, Medium, High), revealing categorical segregation; (d) Heatmap of pairwise differences in mean canopy coverage from Tukey HSD tests, with statistically significant differences marked by asterisks (*)

Bogotá displays a stark pattern of environmental inequality, with tree canopy coverage tightly stratified along socioeconomic lines. As shown in Fig. 3a, neighborhoods in the three lowest socioeconomic strata (strata 1–3) exhibit extremely compressed distributions of canopy cover, with medians near zero, minimal interquartile ranges, and few outliers exceeding 10 %. In contrast, higher strata (strata 4–6) display a clear upward shift in both median and spread, indicating both greater overall canopy presence and increased heterogeneity in greener, wealthier areas.

Population-weighted means further quantify this disparity (Fig. 3b). The lowest strata show negligible average coverage: 0.83 % (stratum 1), 0.58 % (stratum 2), and 0.76 % (stratum 3). A marked inflection appears at stratum 4 (2.21 %), followed by 3.14 % in stratum 5, and a peak of 6.36 % in stratum 6—roughly an eightfold increase from the poorest to the wealthiest areas.

Statistical tests confirm that these differences are highly significant. A one-way ANOVA reveals strong between-group variation ($F_{(5,\,37402)} = 1366.30$, $p < 0.001$). Post-hoc Tukey tests show that stratum 6 differs significantly from all others (mean differences ≈3.64–5.95 pp, all $p<0.001$); stratum 5 exceeds strata 1–4 by 1.13–2.31 pp; and stratum 4 exceeds strata 1–3 by ≈1.07–1.11 pp. Differences between strata 1 and 3 and between 2 and 3 are not significant, while the small gap between 1 and 2 (−0.10 pp) is significant at the 5% level. Non-parametric checks yield the same conclusion (Kruskal–Wallis $H = 8141.53$, $p < 0.001$; Levene’s $W = 1100.25$, $p < 0.001$).

Categorical analysis of canopy cover (Fig. 3c) further highlights this environmental stratification. In strata 1–3, more than 99 % of polygons fall into the “Low” canopy class (<10 %; counts: 6290/62/1, 14898/83/5, 11721/51/2 for Low/Medium/High, respectively). In contrast, Medium canopy accounts for 2.7 % of polygons in stratum 4 (64 of 2418), 6.0 % in stratum 5 (61 of 1022), and 18.3 % in stratum 6 (156 of 855), where High canopy also appears (2.7 %). A chi-square test confirms a strong association between canopy category and socioeconomic status ($\chi ^{2} = 2964.35$, $df = 10$, $p < 0.001$).

Finally, tree canopy coverage is spatially clustered across Bogotá. Moran’s $I = 0.361$ ($z = 142.53$, $p_{\mathrm{sim}} < 0.001$), computed using a queen contiguity graph with $k{=}8$ KNN fallback, indicates strong positive spatial autocorrelation. This suggests that canopy abundance and scarcity are not randomly distributed but instead form contiguous zones that reinforce existing socioeconomic divides, producing a green-rich versus green-poor urban landscape.

##### Summary measures of distributional inequality

Population–weighted inequality metrics corroborate the stark stratification described above. The Canopy Concentration Index (CCI) for Bogotá is 0.436, signalling a strong pro-rich bias: tree canopy is disproportionately concentrated among the wealthiest population groups. The weighted Gini coefficient is 0.814, a level considered “extreme” even in income analyses and well above values typically reported for urban environmental amenities.

To separate inequality attributable to socioeconomic grouping from residual dispersion, we computed the decomposable Theil T index. The overall Theil T is 1.404; of this, 0.369 (26.3 %) arises *between* strata, while 1.034 (73.7 %) reflects inequality *within* strata. Similarly, the Theil L (mean log deviation) is 6.783, with 0.334 (4.9 %) between-group and 6.449 (95.1 %) within-group. While Figs. 3a–c highlight clear average differences across strata, many polygons within higher strata also exhibit very low canopy. This long lower tail inflates within-stratum dispersion, explaining why the decomposition attributes most inequality to the within-group component rather than solely to between-group gaps. These results show that while socioeconomic stratification explains a large share of canopy inequality, even within individual strata canopy distribution is highly uneven.

**Figure 4:**
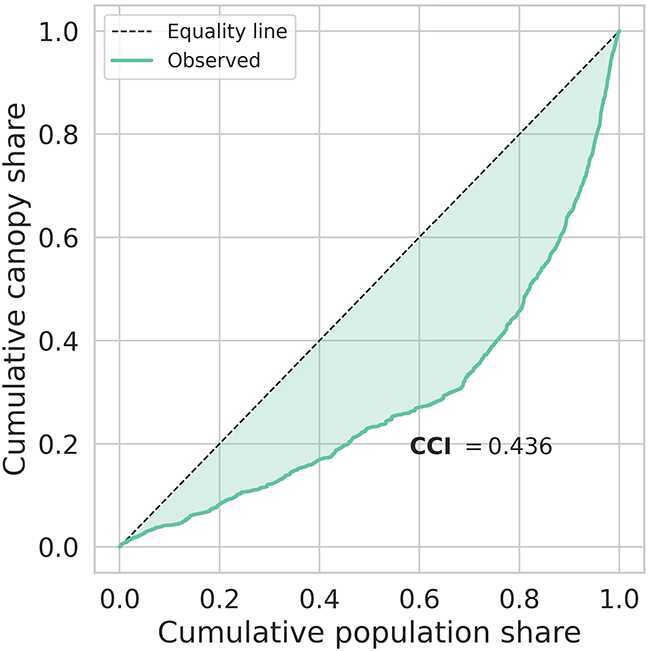
Concentration curve for population-weighted tree-canopy coverage in Bogotá. The shaded area between the observed distribution (blue) and perfect equity (dashed) corresponds to the Canopy Concentration Index (CCI = 0.436)

These findings are visualised in the concentration curve (Fig. 4), where the observed distribution of cumulative canopy share lies far below the equality line. The shaded gap represents the CCI and graphically reinforces the magnitude of Bogotá’s environmental disparity.

#### Bogotá: disparities in access to public green space

We applied an OSM workflow to quantify walking-distance access to public parks in Bogotá. The city-wide inequality metrics are moderate—the weighted Gini for nearest-park distance is 0.360 and the Theil T is 0.216.

Disaggregating by stratum (Table 2) reveals a gentle upward gradient: the poorest strata (1–2) display the lowest internal dispersion (Gini ≈ 0.34), inequality rises through the middle tiers, and peaks in stratum 5 (Gini = 0.393, Theil T = 0.252) before easing slightly in stratum 6. In other words, park access is on average more even than tree-canopy cover, yet measurable differences remain and are most pronounced in upper-middle-income neighborhoods.

**Table 2:** Population-weighted Gini coefficient and Theil T index for distance to the nearest public park, by stratum in Bogotá

Estrato	Gini coefficient	Theil T index
1	0.338	0.184
2	0.339	0.192
3	0.355	0.207
4	0.362	0.216
5	0.393	0.252
6	0.378	0.242

Two patterns in the distribution help explain these findings. First, lower-income areas tend to have a dense street grid with numerous pocket parks, keeping distances short for a large share of residents and narrowing the spread. However, proximity alone may not reflect equitable access: research in other contexts has shown that parks in lower-income neighborhoods often have fewer amenities, poorer maintenance, and lower perceived safety than those in wealthier areas [23]. Our distance-based metric does not capture such qualitative disparities, suggesting that the apparent equity in park proximity may overstate functional access for lower-income residents. Second, the higher strata combine blocks that sit directly adjacent to large public green areas with blocks located several hundred metres away, producing a long right-hand tail in the distance distribution and elevating inequality metrics even though aggregate green space is plentiful. The relatively small spread across strata (a Gini range of only 0.055) nonetheless indicates that Bogotá’s public-park network is more evenly accessible than its canopy resource, underscoring the value of analysing multiple green-infrastructure layers when assessing environmental equity.

#### Medellín: complex patterns and moderated inequality

**Figure 5:**
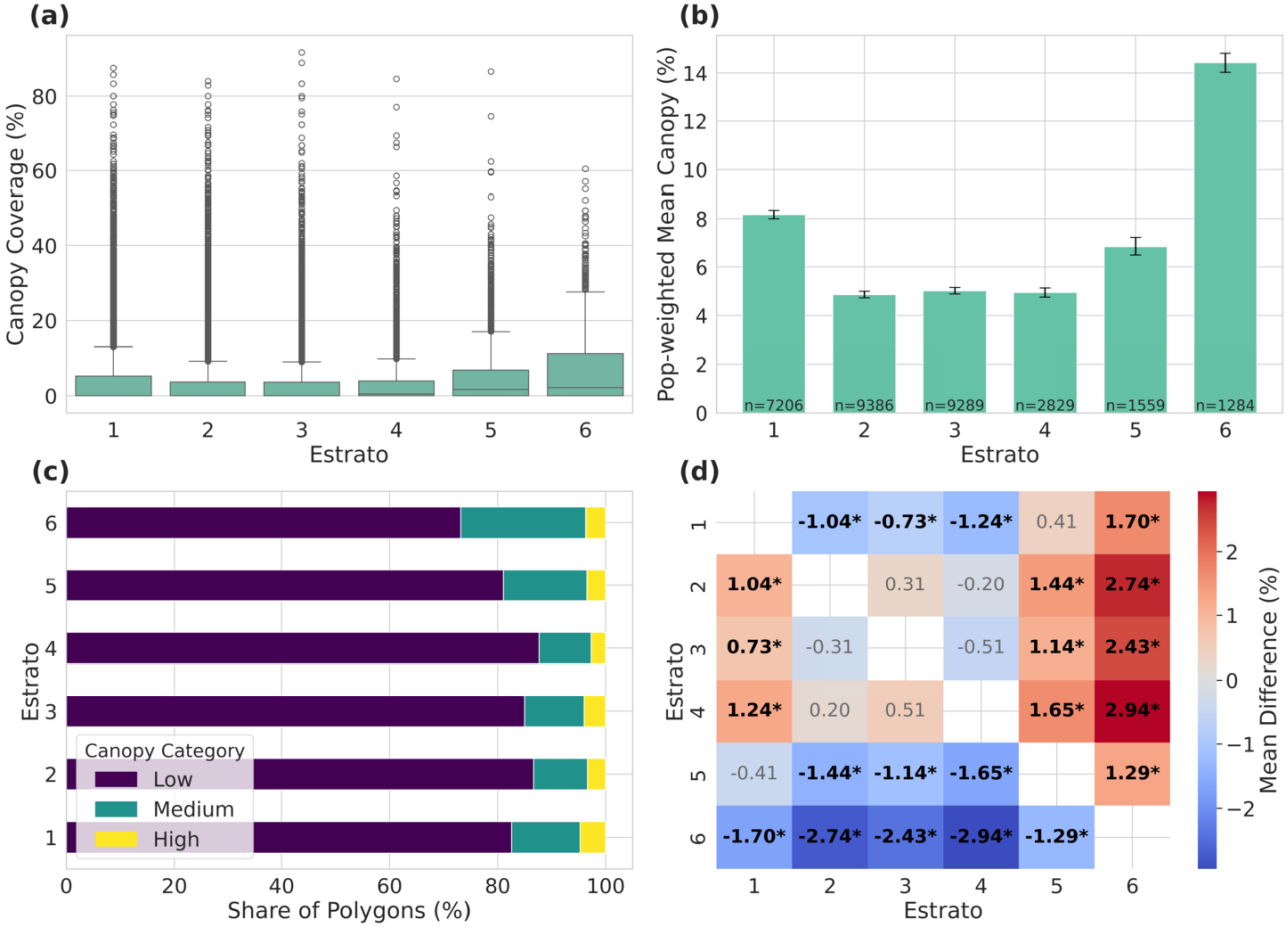
Distribution of tree-canopy cover by socioeconomic stratum in Medellín. (a) Boxplots of canopy coverage (%) by stratum, showing medians, interquartile ranges, and outliers; (b) Mean canopy coverage with 95% confidence intervals by stratum, with *n* indicating the number of spatial units in each group; (c) Stacked bars showing the percentage of area in each canopy class (Low, Medium, High), revealing categorical segregation; (d) Heatmap of pairwise differences in mean canopy coverage from Tukey HSD tests, with statistically significant differences marked by asterisks (*)

Medellín’s urban forest exhibits higher baseline canopy and more moderate inequality than Bogotá. As shown in Fig. 5a, all strata have non–zero medians with substantial overlap, indicating a compressed inequality structure relative to the capital.

Population–weighted means further quantify this pattern (Fig. 5b): 8.17 % (stratum 1), 4.86 % (stratum 2), 5.02 % (stratum 3), 4.95 % (stratum 4), 6.83 % (stratum 5), and 14.43 % (stratum 6). The profile is shallowly U–shaped: the lowest tier is relatively green, the middle tiers (2–4) cluster around ∼5%, and the wealthiest tier carries a clear premium.

Statistical tests confirm that these differences, while smaller than in Bogotá, are significant. A one–way ANOVA reports strong between–group variation ($F_{(5,\,31547)} = 29.21$, $p < 0.001$), and the rank association is weak but positive (Spearman $\rho = 0.081$, $p < 0.001$). Post–hoc Tukey tests show stratum 6 higher than all others (mean differences ≈1.29–2.94 pp, all $p<0.01$); stratum 5 exceeds strata 2–4 by 1.14–1.65 pp; and stratum 1 exceeds strata 2–4 by 0.73–1.24 pp. Several pairwise differences, such as between strata 2 and 3 or between 1 and 5, are not significant. Non–parametric checks reach the same conclusion (Kruskal–Wallis $H = 500.0$, $p < 0.001$; Levene’s $W = 25.5$, $p < 0.001$).

Categorical analysis (Fig. 5c) underscores this moderated gradient. Even the poorest tier records 20.3 % of polygons in Medium + High canopy (917 and 338 of 7206, respectively), and every stratum contains a visible medium–canopy segment. In the wealthiest tier, Medium canopy accounts for 23.1 % of polygons (297 of 1284) and High canopy for 3.7 %. A chi–square test confirms that canopy category distribution is significantly associated with socioeconomic status ($\chi ^{2} = 270.6$, $df = 10$, $p < 0.001$), though the effect size is far smaller than in Bogotá.

Finally, spatial autocorrelation is modest (Moran’s $I = 0.176$, $z = 35.8$, $p_{\mathrm{sim}} = 0.001$), consistent with a mosaic of greener and barer blocks rather than large homogeneous zones of deprivation or privilege.

##### Summary measures of distributional inequality

The same inequality diagnostics applied to Medellín confirm a markedly more egalitarian canopy distribution than that observed for Bogotá. The CCI is just 0.134, signalling only a mild pro-rich neighborhood tilt. The population-weighted Gini coefficient is 0.584, well below Bogotá’s 0.814 and consistent with the higher baseline canopy visible across lower-income areas.

The decomposable Theil T index offers a complementary view. The total Theil T is 0.594, of which only 0.082 (13.8 %) is attributable to *between-stratum* differences, while 0.513 (86.2 %) arises *within* strata. Similarly, the Theil L (mean log deviation) is 1.928, with just 0.069 (3.6 %) between-group and the remaining 1.859 (96.4 %) within-group. Thus, residual inequality is driven largely by local heterogeneity rather than systematic socioeconomic segregation.

**Figure 6:**
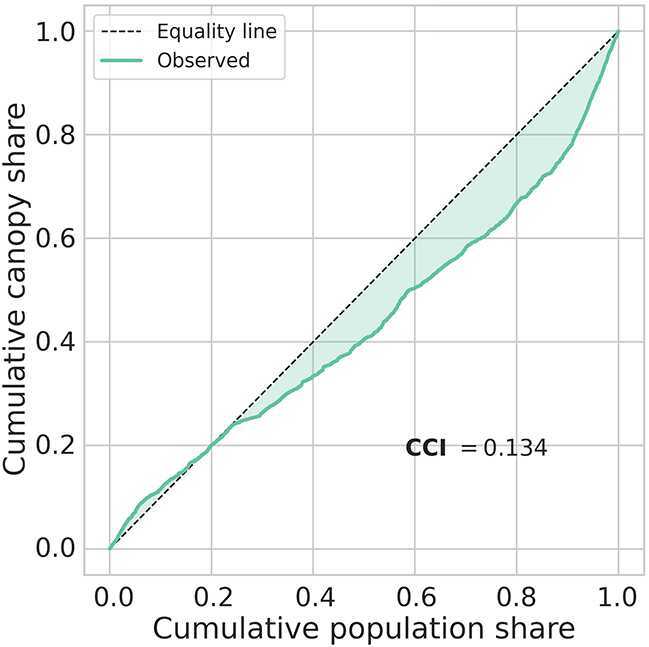
Concentration curve for population-weighted tree-canopy coverage in Medellín. The shaded area between the observed distribution (blue) and the line of perfect equity (dashed) yields the Canopy Concentration Index (CCI = 0.134)

Figure 6 visually reinforces these findings: the concentration curve lies close to the equality line for most of the population, with only a modest bow in the upper-income tail. Together, the statistics and the graphic demonstrate that, while Medellín’s urban greenery is not perfectly equal, extreme deprivation is rare and broad sections of the city enjoy canopy shares close to their population shares—a stark contrast to the sharp environmental stratification documented in Bogotá.

#### Medellín: disparities in access to public green space

To complement the canopy analysis, we assessed equity in access to formal public parks extracted from OSM. Access was defined as Euclidean distance from every residential block centroid to the nearest mapped park, and population-weighted inequality was summarised with the Gini coefficient and Theil T index.

City-wide inequality is moderate: the weighted Gini for distance equals 0.387 and the Theil T is 0.250. Both values sit well below the canopy-based metrics, confirming that the public-park network is spatially more egalitarian than overall vegetative cover. Equity, however, is not uniform across the social gradient. Table 3 shows a shallow U-shape: the middle-income tier (stratum 3) enjoys the lowest inequality, while both the poorest and the wealthiest ends post slightly higher—but still only moderate—dispersion. Importantly, differences among strata are small: the distance Gini varies by just 0.06 across the entire socioeconomic spectrum, and the between-stratum component of the Theil T is also very small.

**Table 3:** Population-weighted Gini coefficient and Theil T index for distance to the nearest public park, by stratum in Medellín

Estrato	Gini coefficient	Theil T index
1	0.383	0.244
2	0.391	0.247
3	0.333	0.177
4	0.372	0.223
5	0.377	0.245
6	0.384	0.228

Two key insights emerge.

1. **A persistent floor effect for the poor.** The two lowest strata exhibit the highest internal inequality in park access: stratum 2 has the largest Gini (0.391) and Theil T (0.247), while stratum 1 follows closely (Gini 0.383, Theil T 0.244). This points to a long tail of blocks that remain far from a public park—an equity gap invisible in citywide averages but consequential for low-income residents.
2. **Apparent diminishing returns at the top.** Stratum 6 shows a Gini coefficient (0.384) nearly identical to stratum 1, though for the Theil T, stratum 5 (0.245) is most similar to stratum 1. This occurs for a different reason than in lower strata. Our metric only considers public parks. In wealthy areas, many households already enjoy large private gardens or access to gated green spaces, which are not captured in OSM. As a result, some blocks arguably record long distances to public parks even though residents are not reliant on them, artificially inflating intra-class variance. In practice, this inequality is less critical than in Stratum 1, where public parks are often the only source of greenery.

### High-risk zones and spatial disparities

#### Defining high-risk populations

We identify “high-risk” polygons as areas experiencing a dual burden: low canopy coverage ($C_{p} < 10\%$) combined with high population density ($P_{p} > T$). This definition captures neighborhoods where large numbers of residents face limited access to urban green infrastructure. The population threshold *T* serves as a policy-relevant filter, ensuring that flagged areas represent substantial concentrations of affected residents rather than sparsely populated zones with incidental low canopy coverage.

#### Sensitivity analysis of population thresholds

To assess the robustness of our findings, we varied the population threshold across $T \in \{400, 500, 600\}$ and tracked four key diagnostics: (i) the number of flagged polygons, (ii) the total population living in flagged areas, (iii) the proportion of all low-canopy polygons that qualify as high-risk, and (iv) the share of each city’s population residing in high-risk zones. We also computed Jaccard similarity indices between successive thresholds to quantify spatial stability.

In Bogotá, increasing *T* from 400 to 600 reduces flagged polygons from 4,219 to 1,908, with the exposed population declining from 3.25 million to 2.14 million. The proportion of low-canopy polygons classified as high-risk drops from 11.1% to 5.0%, while the share of the city’s population living in these zones decreases from 55.8% to 36.8%. Despite these quantitative changes, the spatial footprint remains stable (Jaccard similarity = 0.645 for $T = 400$ vs. $T = 500$, and 0.701 for $T = 500$ vs. $T = 600$). The threshold $T = 500$ corresponds approximately to the 93rd percentile of polygon populations, effectively targeting the largest residential blocks while maintaining analytical tractability.

In Medellín, the pattern is similar but with different magnitudes. Flagged polygons decrease from 934 (at $T = 400$) to 441 (at $T = 600$), with the exposed population falling from 0.96 million to 0.72 million. The proportion of low-canopy polygons flagged declines from 3.4% to 1.6%, and the citywide population share drops from 44.0% to 33.1%. Spatial stability is comparably high (Jaccard similarity = 0.662 for $T = 400$ vs. $T = 500$, and 0.714 for $T = 500$ vs. $T = 600$). Here, $T = 500$ represents approximately the 97th percentile of polygon populations, reflecting Medellín’s different urban density structure.

These sensitivity analyses demonstrate that while absolute numbers vary with threshold selection, the spatial concentration of high-risk areas and the fundamental policy implications remain consistent. We adopt $T = 500$ as our primary threshold, balancing the need to identify areas with substantial affected populations while maintaining comparability across both cities.

#### Spatial distribution of high-risk zones

**Figure 7:**
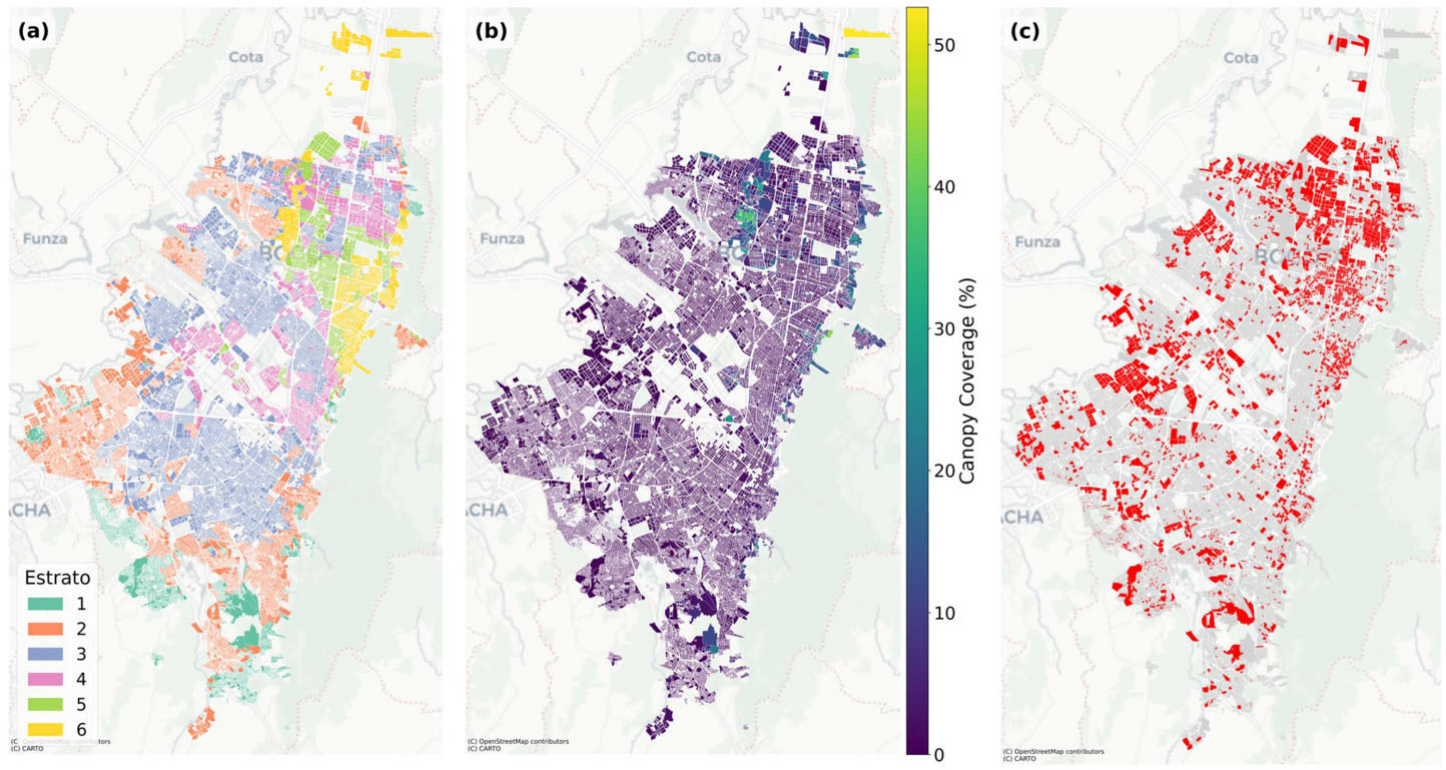
Bogotá spatial patterns: (a) socioeconomic strata (*estratos*); (b) canopy coverage (%); (c) high-risk polygons ($C_{p} < 10\%$ and $P_{p} > 500$; red). High-risk areas form distinct spatial clusters concentrated in the southern, southwestern, and western portions of the city, coinciding with zones of low canopy coverage and predominantly lower socioeconomic strata

**Figure 8:**
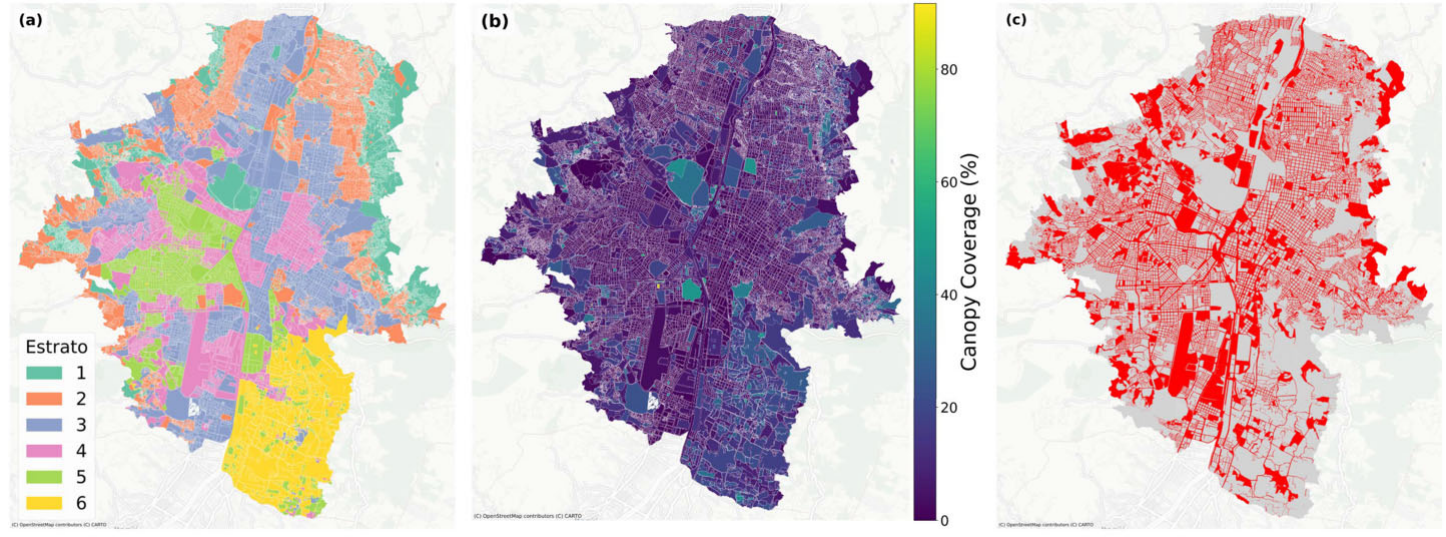
Medellín spatial patterns: (a) socioeconomic strata; (b) canopy coverage (%); (c) high-risk polygons ($C_{p} < 10\%$ and $P_{p} > 500$; red). High-risk areas exhibit a more fragmented distribution compared to Bogotá, with concentrations primarily in central urban zones where low canopy coverage intersects with lower socioeconomic strata

Figures 7 and 8 reveal markedly different spatial patterns of environmental risk between the two cities. In Bogotá (Fig. 7), high-risk polygons form several distinct spatial clusters rather than random distributions. The most prominent concentrations appear in the southern, southwestern, and western portions of the city, creating what can be characterized as contiguous zones of environmental disadvantage. These areas correspond closely to the lowest canopy coverage zones visible in panel (b) and align predominantly with lower socioeconomic strata (estratos 1-3) shown in panel (a).

Medellín presents a contrasting spatial structure (Fig. 8). High-risk polygons exhibit a more fragmented pattern, with concentrations primarily in the central urban core and select peripheral areas. Unlike Bogotá’s extensive clustered zones, Medellín’s high-risk areas appear as smaller, more dispersed pockets. This fragmentation likely reflects the city’s mountainous topography, which creates a more complex relationship between urban density, canopy coverage, and socioeconomic stratification.

#### Bivariate spatial analysis

**Figure 9:**
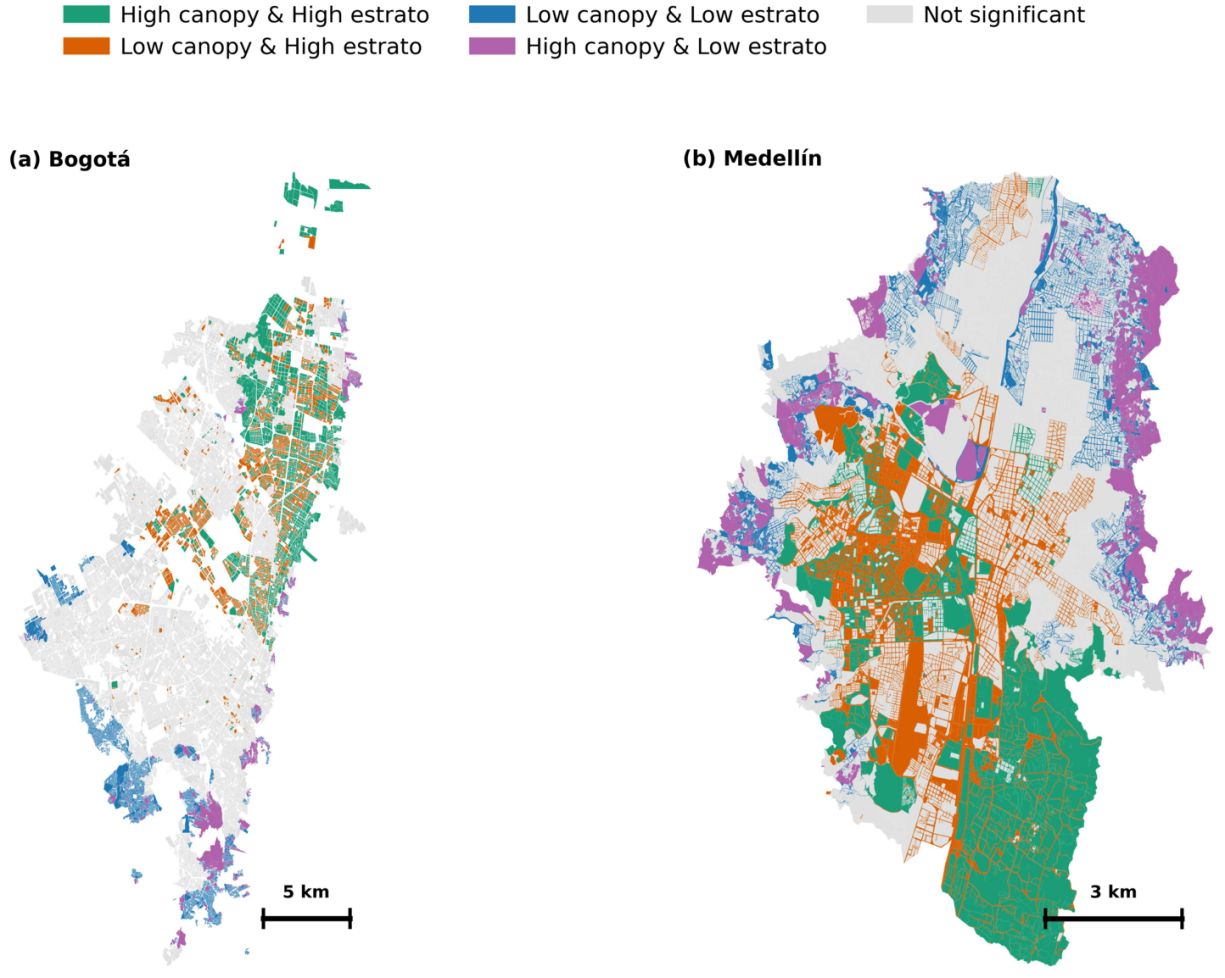
Bivariate LISA clusters linking canopy coverage and socioeconomic status. Green: high canopy & high *estrato* (HH); orange: low canopy & high *estrato* (LH); blue: low canopy & low *estrato* (LL); purple: high canopy & low *estrato* (HL); gray: not significant (5% permutations). Panels: (a) Bogotá, (b) Medellín

To move beyond visual inspection, we computed bivariate Local Indicators of Spatial Association (LISA) between canopy coverage and *estrato* to identify statistically significant spatial clusters. Figure 9 classifies polygons into four significant cluster types (5% significance level): High canopy/High *estrato* (HH), Low canopy/High *estrato* (LH), Low canopy/Low *estrato* (LL), and High canopy/Low *estrato* (HL), with non-significant areas shown in gray.

In Bogotá (panel a), the bivariate LISA analysis reveals a relatively fragmented pattern with limited areas of significant spatial clustering. The northern portion shows scattered clusters of High canopy/High *estrato* (HH, green) interspersed with Low canopy/High *estrato* (LH, orange) areas, suggesting that even within affluent zones, green space distribution is heterogeneous. Low canopy/Low *estrato* (LL, blue) clusters appear in the southern periphery, corresponding to areas identified as high-risk in our previous analysis. High canopy/Low *estrato* (HL, purple) areas are distributed across various locations throughout the city. The predominance of non-significant areas (gray) indicates that much of Bogotá does not exhibit strong spatial clustering of the canopy-*estrato* relationship.

Medellín (panel b) displays a more pronounced and interpretable spatial structure. The eastern periphery is dominated by extensive High canopy/Low *estrato* (HL, purple) clusters, reflecting the mountainous terrain where lower-income populations reside in areas with abundant natural vegetation. The central urban core shows a clear concentration of Low canopy/High *estrato* (LH, orange) areas, indicating that wealthy central neighborhoods experience limited tree cover despite their economic advantages. High canopy/High *estrato* (HH, green) clusters appear in specific zones, particularly in the southern and southeastern sections. Low canopy/Low *estrato* (LL, blue) areas are scattered throughout the urban fabric, often corresponding to the high-risk zones identified in our analysis.

## Discussion

This study reveals how the spatial distribution of urban greenery—both in the form of tree canopy and access to public parks—remains deeply patterned by socio-economic stratification. Across two of Colombia’s most prominent cities, we find that the environmental, health, and social benefits of greenery are not equally shared. But more importantly, we show that these disparities are measurable, interpretable, and—crucially—actionable using open data and reproducible tools.

In Bogotá, greenery is heavily concentrated in affluent neighborhoods. The wealthiest strata enjoy not only significantly more tree canopy but also greater internal heterogeneity, combining exclusive green enclaves with large private plots and access to public parks. At the other end of the spectrum, lower-income neighborhoods are systematically deprived of canopy cover, with the majority falling into the lowest canopy category. This sharp vertical gradient is not merely cosmetic—it reflects and reinforces unequal exposure to urban heat, pollution, and the psychological and physical health burdens of environmental scarcity. These inequalities persist despite formal planning efforts and sustainability rhetoric, revealing a disconnect between policy aspirations and spatial outcomes.

Medellín, by contrast, offers a more complex and moderated profile. While inequality still exists, it is notably attenuated. Canopy cover is more widespread across socio-economic groups, and park access is more evenly distributed. The city’s history of integrated urban interventions—including its Green Corridors program [33, 34]—may partly explain this relative equity, alongside a more compact and topographically varied urban form that limits the consolidation of privilege. Still, subtle patterns persist: both the poorest and richest strata show tails of limited access. These findings caution against assuming that equitable green distribution follows automatically from aggregate increases in green supply.

While these differences may be partly explained by planning choices and urban evolution, the impact of the distinctive eco-geographic profiles of both locations cannot be ignored. Bogotá sits on a high-altitude savanna-wetland mosaic, where the continuous forest canopy was limited to rivers corridors and surrounding mountain slopes, while much of the Bogotá plain was open grassland and *páramo* shrubs [108]. Consequently, most of the tree canopy on the plain is the result of deliberatively planting. By contrast, Medellín occupies a lower humid Andean valley that used to be covered by dense tropical humid pre-montane forest with a closed canopy [109]. In this case, urban expansion often replaces forest rather than open grassland, and the climate conditions allows vegetation to regenerate quickly, even in informal or neglected areas. These biophysical preexistences interact with socio-spatial urban processes: while in Bogotá, the green inequities can be explained mostly in a context where the canopy is basically a human-constructed feature, more dependent on purposely action and intent; in Medellín, the ecological baseline eases the retention and growth of spontaneous canopy, resulting in a different spatial pattern of inequality more distributed across the city.

Topography further conditions how greenery is distributed and accessed in both cities. In Medellín, informal and lower-income households are disproportionately located on steep, landslide-prone slopes (*laderas*) scattered across the city, where unmanaged vegetation often persists as spontaneous regrowth on marginal land and contributes to slope stabilization and microclimate regulation [110–113], though its quality and accessibility remain uneven. By contrast, in Bogotá the flatter topography reduces slope-related risks, but green inequities emerge from the uneven distribution of deliberately planted canopy and the concentration of large parks in wealthier districts [114]. Together, these differences underscore how physical geography, risk exposure, and ecosystem services interact with socio-spatial processes to shape distinct patterns of urban green inequality. The high-risk zone analysis reinforces this, revealing that environmental burdens are not randomly distributed but concentrate in specific areas, creating distinct geographies of vulnerability that require context-specific policy responses.

Crucially, our results challenge dominant notions of what constitutes a “smart” or “sustainable” city. Too often, urban performance is assessed through city-wide averages or technocentric indices that prize digital infrastructure and ecological footprints without asking who benefits. A city can score well on green space per capita while maintaining vast inequalities in who has access to that space. By shifting the unit of analysis to the block or neighborhood and weighting by population, our approach reveals inequities that aggregated metrics obscure. Environmental justice is not a byproduct of optimization, it must be measured directly. Our findings resonate strongly with how some of the leading smart city indices look at urban greenery, which shows that greenery is almost universally measured as a single, citywide value—most often as green space per capita—without consideration of spatial distribution, accessibility, or canopy quality. None of the reviewed frameworks provides sub-city disaggregation. In socio-spatially stratified cities like Bogotá, this aggregation can mask profound inequities: the affluent enjoy abundant, well-maintained greenery while low-income neighborhoods remain deprived, yet both realities collapse into the same average. Such blind spots are not incidental—they are embedded in the way “smart” performance is operationalized. Our block-level, population-weighted indicators directly address this gap, translating equity from an aspirational principle into a measurable, comparable, and policy-relevant dimension of smart city performance.

Methodologically, this study demonstrates the power of open-source, high-resolution analysis. Our geospatial pipeline, now implemented in the greenR R package [90], integrates multiple data sources into a scalable and transparent framework. The canopy-height modelling function adds critical functionality to assess vertical green infrastructure using widely available satellite data. While this paper focuses on Bogotá and Medellín, two cities where stratified socio-economic data are publicly available, the workflow can be extended to any urban area where relevant data exist. In this way, the tools serve as a foundation for building more context-aware and justice-oriented green planning systems across the Global South and beyond.

One of the fundamental strengths of this study lies in its broader and more inclusive consideration of urban greenery. Whereas many previous analyses focus exclusively on formally designated green areas or public parks, we incorporate both OSM green-related features and high-resolution tree canopy cover to provide a more holistic and nuanced assessment. This approach reduces the inherent limitations of incomplete mapping and records in informal settlements and other marginalized areas, which is a persistent challenge in the Global South [115]. By combining mapped green features with tree cover, we capture distinctive, complementary, and at times overlapping forms of urban greenery, each contributing in different ways to ecosystem services, microclimate regulation, and social well-being [116, 117].

From an Urban Political Ecology perspective, this methodological approach challenges political and institutional biases embedded in official inventories, which tend to exclude informal settlements. Recognizing that green infrastructure is socially produced and politically distributed, we question official planning practices that may inadvertently reproduce environment injustices rather than address them [33]. From an Environmental Justice standpoint, this work addresses recognitional justice by making visible and highlighting the value of the green infrastructure that supports everyday life in under-represented areas, while also revealing the distributional and procedural inequities in how urban nature is mapped, valued, and governed. By doing so, we move beyond aggregate metrics to produce a more granular, equity-oriented equity-oriented diagnosis of how the different forms of green infrastructure shape urban environment.

That said, we acknowledge that there are some limitations. OSM data may omit informal green spaces, and Euclidean distances simplify complex mobility barriers. Furthermore, gaps in remote sensing or population data often disproportionately affect dense informal settlements; since these areas typically lack greenery, any data omission likely results in a conservative underestimation of the true extent of inequality. These constraints do not diminish the findings but rather suggest that the environmental divide may be even more acute than reported.

This study illustrates how fine-grained, spatially explicit indicators can support more equitable urban planning. By integrating open data sources into a transparent and reproducible workflow, we provide tools that help identify where environmental investments are most needed. These indicators are not ends in themselves, but inputs for a more just and targeted urban sustainability agenda. From heat mitigation to mental health, biodiversity to social cohesion, greenery functions as essential infrastructure. Ensuring its fair distribution is both a challenge of justice and a practical necessity for building more resilient, inclusive cities.

## Conclusion

This study shows that who benefits from urban greenery can be measured with precision and evaluated against explicit equity goals. By integrating canopy height models, population grids, socio-economic strata, and OSM green spaces into a single, reproducible workflow, we moved beyond citywide averages to quantify how both canopy and park access are distributed across people and places, while integrating additional greenery provision not considered in official records. Applied to Bogotá and Medellín, the approach revealed two distinct equity regimes: a strongly stratified canopy landscape in Bogotá versus a more moderate, internally heterogeneous pattern in Medellín, with park access in both cities measurably more even than canopy but still marked by tails of disadvantage. These results underline that the form of greenery matters—vertical cover and proximity to formal parks follow different planning logics and therefore different inequality structures.

Policy implications are clear: equity audits should operate at the same spatial grain as interventions, using population-weighted and distributional metrics rather than single averages. In Bogotá, contiguous clusters of low-canopy, high-population blocks call for large-scale planting and maintenance, while Medellín’s more scattered deficits suit targeted infill and pocket-park strategies. Embedding spatial inequality maps, stratified statistics, and decomposable indices into municipal dashboards can help track whether greening efforts close rather than widen gaps.

To summarize, equitable greenery is achievable only if measured explicitly. The pipeline developed here presents a practical, transparent framework for diagnosing disparities, prioritising investments, and monitoring progress—helping align urban greening with climate resilience, public health, and social justice.

## Data Availability

The data associated with this study are available at https://github.com/sachit27/urban-green-equity-analysis

## Code availability

The code associated with this study is available at https://github.com/sachit27/urban-green-equity-analysis.
